# Synergism of clinical evaluation and penile sonographic imaging in diagnosis of penile fracture: a case report

**DOI:** 10.1186/1752-1947-6-321

**Published:** 2012-09-25

**Authors:** Jibril Oyekunle Bello

**Affiliations:** 1 Urology unit, Department of Surgery, University of Ilorin Teaching Hospital, Ilorin, Nigeria

**Keywords:** Tunica Albuginea, Corpus Cavernosum, Penile fracture, Penile Ultrasound, Sonourethrography

## Abstract

**Introduction:**

Penile fracture is an uncommon urologic emergency, and is the traumatic rupture of the tunica albuginea covering the corpus cavernosa. This usually occurs following blunt trauma sustained during coitus, masturbation or self-manipulations to hide or suppress an erection. Clinical diagnosis can often be easily made with typical history and examination findings. However, the patient may present atypically and/or with a suspicion of associated urethral injury. The roles of various diagnostic investigations are being evaluated in these situations.

**Case presentation:**

We report the case of a 31-year-old African man with penile fracture and suspected associated urethral injury that occurred after self-manipulations to hide an erection.

**Conclusions:**

Penile ultrasound and sonourethrography provide useful additional diagnostic information to supplement clinical history and physical examination findings and can be performed easily, at low cost and with no delays to surgery.

## Introduction

Penile fracture is an uncommon urologic emergency, and is the traumatic rupture of the tunica albuginea (TA) covering the corpus cavernosum (CC). It results when blunt trauma to the erect penis results in a rapid build-up of pressure in the engorged CC, which overwhelms the tensile strength of the TA; this often follows trauma during coitus, masturbation or self-manipulations to hide or suppress an erection [1,2]. Several reports have cast doubts on the value of diagnostic investigations, with some asserting that penile fracture is a clinical diagnosis and others concluding that the investigations add to treatment costs and delays to surgery [2-4]. However, the roles of the various diagnostic investigations are still being evaluated. We report a case of penile fracture with suspected associated urethral injury that had both clinical assessment and inexpensive radiologic evaluations, which together were helpful in making a definitive diagnosis with no delay of surgery.

## Case presentation

**Figure 1:**
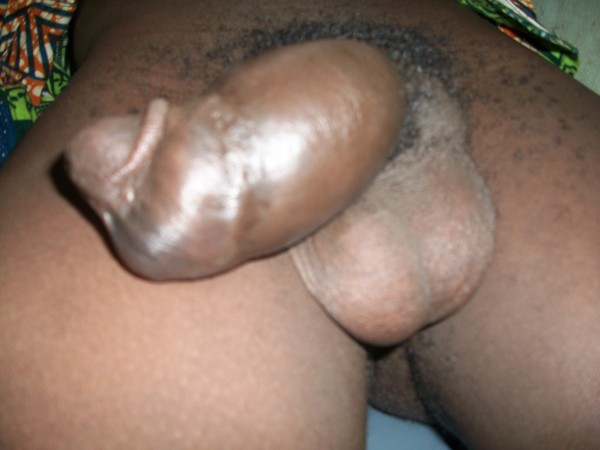
**Penile fracture.** Clinical photograph showing a swollen penis with ‘egg plant’ deformity and deviation to the right side.

**Figure 2:**
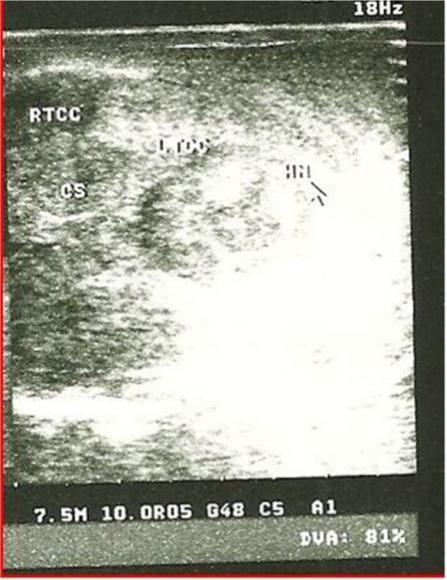
**Penile ultrasound of the proximal third of the penile shaft.** The penile ultrasound was performed an hour after presentation and shows a normal right corpus cavernosum, a ruptured left corpus cavernosum and associated hematoma. The corpus spongiosum appears normal.

**Figure 3:**
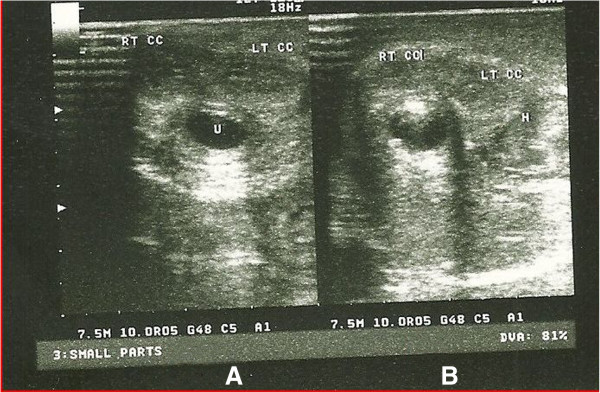
**Sonourethrogram of penile fracture.** (**A**) Sonourethrogram at the distal aspect of the penile shaft, showing a normal right and left corpus cavernosa and normal urethra. (**B**) Sonourethrogram at the proximal third of the penile shaft showing a normal right corpus cavernosum, a ruptured left corpus cavernosum with associated hematoma. On real-time imaging the urethra appears intact. This sonourethrogram was performed immediately following the penile ultrasound. The urethra was distended retrogradely by instilling normal saline via a Foley catheter placed at the fossa navicularis.

A 31-year-old African man presented to our emergency unit with a four-hour history of penile swelling and voiding difficulty. He had earlier tried to suppress an erection before his routine morning religious rituals by kneading on his erect penis. He heard a popping sound with associated minimal pain and sudden detumescence. He noticed no bleeding per urethra but subsequently had difficulty with voiding and urinary frequency. On physical examination, he had a grossly swollen and deformed penis with deviation to the right side (Figure 1). A diagnosis of penile fracture with probable associated urethral injury was made. Bedside urine analysis results were negative for blood and he was taken for penile ultrasound and sonourethrography about an hour after presentation to the emergency unit. This was performed using a high-frequency 7.5MHz transducer with our patient in a supine position and the penis supported with towels between the thighs. A generous amount of sonographic acoustic gel was applied on the dorsum of the penis and images were obtained both in longitudinal and transverse planes beginning at the glans and proceeding proximally to the root of the penis. This was performed initially without urethral distension. A 16Fr Foley catheter was then placed at the urethral meatus with its balloon inflated with 2mL of water at the fossa navicularis. Retrograde urethral distension using normal saline was achieved via the catheter and sonographic images were obtained as above. The penile ultrasound showed a tear of the TA covering the ventrolateral aspect of left CC with associated hematoma in the proximal third of the penile shaft, and the real-time sonourethrography additionally revealed an intact urethra (Figures 2 and 3A,B). He also had retrograde urethrography performed, which also showed an intact urethra (Additional file 1). Three hours after presenting to the hospital he had penile surgery via a subcoronal circumferential degloving incision. A Foley urethral catheter was placed pre-operatively to identify and avoid injury to the urethra. A 1.5cm tear of the TA in the ventrolateral aspect of the proximal third of the left CC just adjacent to the urethra was seen (Additional file 2). The hematoma was evacuated and the tear repaired with 2/0 silk with burying of the knots. An artificial erection created with intra-cavernosal injection of normal saline revealed an intact repair with no leakages. The wound was closed with 3/0 vicryl (Additional file 3). He was discharged 24 hours after surgery and advised to abstain from sexual activity for six weeks. At one-week follow-up, he reported return of good nocturnal erections and normal voiding.

## Discussion

Blunt trauma to the erect penis may generate pressures in excess of 1500mmHg within the CC, resulting in rupture of the TA [1]. The TA thins out to 0.25mm during erection from a thickness of 2mm in the flaccid state and is incapable of withstanding this enormous build-up of pressure in the CC [1]. Such trauma may occur during coitus with the penis slipping out of the vagina and hitting the pubis or the rigid perineum [1,2]. Masturbations, self-manipulations and kneading of the penis to achieve or hide an erection have also been reported to result in penile fractures [1,2].

The patient often reports a popping sound, mild to severe pain and sudden detumescence [1,2]. This is followed by swelling of the penis, which may involve the scrotum and or the lower abdominal wall depending on the integrity or otherwise of the Buck’s fascia. Physical examination typically reveals swelling and bruising of the penis (egg plant deformity), deviation of the penis from the side of rupture and palpable subdartos hematoma (rolling sign) [1]. A clinical diagnosis can often be easily made with the typical history and physical examination findings described [3]. The patient may, however, present atypically, reporting no preceding traumatic events and or no pain, no popping sounds or swellings and physical examination findings may be unremarkable or confusing [5,6]. Patients with urethral injury may additionally give a history of bleeding per urethra, difficulty with or inability to micturate and, in those presenting late, signs of urinary extravasation may be observed [7-9]. Our patient gave a history of difficulty with voiding and increased frequency of micturition, which led to the clinical suspicion of an associated urethral injury.

Radiologic evaluations have been described for patients with penile fracture and these include retrograde urethrography, cavernosography, magnetic resonance imaging (MRI), penile ultrasound and sonourethrography [5-9]. Retrograde urethrography and cavernosography are invasive, and significant false negative findings have been reported, while MRI is an excellent imaging modality but expensive and not widely available [7-10]. Penile ultrasound is simple, non-invasive and useful in detecting the site and side of rupture in the TA and the associated hematoma while sonourethrography may show a hematoma of the corpus spongiosum and/or extravasation of fluid media (used in distending the urethra), which are indicative of urethral injury [6,9]. While we found no study describing the sensitivity or specificity of these sonographic evaluations in penile fracture, sonourethrography for urethral injury following coital trauma has been described and was able to detect a hematoma in the corpus spongiosum of the penile urethra, identifying an injury which was missed by both retrograde urethrography and penile ultrasonography (performed without urethral distension) [10]. This suggests sonourethrography may be useful in diagnosis of penile fractures with suspected urethral injury.

Our patient had both penile ultrasound and sonourethrography performed in the same sitting about an hour after presentation to the emergency unit. This was a simple, non-time-consuming procedure, and inexpensive at a cost of US$7. It identified the area of tear in the TA with the associated hematoma and ruled out urethral injury as there was no evidence of hematoma seen in the corpus spongiosum or extravasation of the fluid used in distending the urethra during real-time sonourethrography. Our patient additionally had retrograde urethrography, which also revealed no urethral injury but unlike the sonourethrogram was associated with radiation exposure, was more invasive with risks of allergic reaction to contrast and gave no information about the tear in TA or associated hematoma. It was also six times the cost of the sonourethrogram and penile ultrasound put together.

Early surgery for penile fracture has been advocated to prevent the significantly greater complications seen with delayed surgery [1-3]. Our patient had surgery three hours after his presentation to the emergency unit, incurring no delay to surgery from the radiological investigations performed.

## Conclusions

Evaluation of penile fracture by penile ultrasound and sonourethrography gives additional useful diagnostic information to that obtained from clinical history and examination in patients with atypical presentations and or suspected associated urethral injury. The penile ultrasound and sonourethrography can be performed easily, at relatively low cost and results in no delays to surgery.

## Consent

Written informed consent was obtained from the patient for publication of this case report and any accompanying images. A copy of the written consent is available for review by the Editor-in-Chief of this journal.

## Competing interests

The author declares that there are no competing interests.
